# Supplementary material for: Droplet-based microfluidics platform for investigation of protoplast development of three exemplary plant species
Source: Sci Rep. 2025 Nov 18;15:40332. doi: 10.1038/s41598-025-28956-w (PMC12627680; doi:10.1038/s41598-025-28956-w)

Supplementary information for the research paper ‘Droplet-based microfluidics platform for investigation of protoplast development of three exemplary plant species’

Supplementary Table S1. Media composition and solutions sources used during protoplasts isolation and culture.

| Solutions | Manufacturer | Media | | | | | | | |
| --- | --- | --- | --- | --- | --- | --- | --- | --- | --- |
|  |  | Preplasmolysis solution | BNE9 | Washing solution | Culture medium 8pm7 | MMM | F-PIN | Culture medium F-PCN |  |
| Sorbitol | Duchefa | 0.3 M |  |  | 0.25 g·L^-1^ |  |  |  |  |
| Glycine | Duchefa | 0.1 M |  |  |  |  |  |  |  |
| CaCl_2_·2H_2_O | VWR | 0.05 M | 0.6 g·L^-1^ | 44.2 g·L^-1^ |  |  | 0.64 g·L^-1^ |  |  |
| NH_4_NO_3_ | Grüssing |  | 0.6 g·L^-1^ | 0.16 g·L^-1^ | 1.65 g·L^-1^ |  |  |  |  |
| KH_2_PO_4_ | VWR |  | 0.17 g·L^-1^ | 0.136 g·L^-1^ | 0.17 g·L^-1^ |  | 0.17 g·L^-1^ |  |  |
| KCl | Roth |  | 0.3 g·L^-1^ | 18.8 g·L^-1^ | 0.3 g·L^-1^ |  |  |  |  |
| Sucrose | Duchefa |  | Approximately 125 g·L^-1^ | Approximately 23 g·L^-1^ | Approximately 125 g·L^-1^ |  | Approximately 130 g·L^-1^ | Approximately 20 g·L^-1^ |  |
| Glucose | Applichem |  |  |  |  |  |  | 80 g·L^-1^ |  |
| KNO_3_ | Merck |  | 1.9 g·L^-1^ |  | 1.9 g·L^-1^ |  | 1.012 g·L^-1^ | 1.012 g·L^-1^ |  |
| MgSO_4_·7H_2_O | VWR |  | 0.6 g·L^-1^ |  | 3.7 g·L^-1^ | 1.25 g·L^-1^ | 0.37 g·L^-1^ | 0.37 g·L^-1^ |  |
| MgCl_2_·6H_2_O | VWR |  |  |  |  | 1.02 g·L^-1^ |  |  |  |
| KI | Roth |  |  |  | 0.83 mg·L^-1^ |  | 0.83 mg·L^-1^ | 0.83 mg·L^-1^ |  |
| H_3_BO_3_ | Roth |  |  |  | 6.2 mg·L^-1^ |  | 6.2 mg·L^-1^ | 6.2 mg·L^-1^ |  |
| MnSO_4_·4H_2_O | Geyer |  |  |  | 26.6 mg·L^-1^ |  | 26.6 mg·L^-1^ | 26.6 mg·L^-1^ |  |
| ZnSO_4_·7H_2_O | VWR |  |  |  | 8.6 mg·L^-1^ |  | 8.6 mg·L^-1^ | 8.6 mg·L^-1^ |  |
| Na_2_MoO_4_·2H_2_O | ThGeyer Chemsolute |  |  |  | 0.25 mg·L^-1^ |  | 0.25 mg·L^-1^ | 0.25 mg·L^-1^ |  |
| CuSO_4_·5H_2_O | Merck |  |  |  | 25 µg·L^-1^ |  | 25 µg·L^-1^ | 25 µg·L^-1^ |  |
| CoCl_2_ | Acros |  |  |  | 13.5 µg·L^-1^ |  | 13.5 µg·L^-1^ | 13.5 µg·L^-1^ |  |
| NaFeEDTA | Roth |  |  |  | 36.7 mg·L^-1^ |  | 36.7 mg·L^-1^ | 36.7 mg·L^-1^ |  |
| Myo-inositol | Merck |  |  |  | 100 mg·L^-1^ |  | 200 mg·L^-1^ | 200 mg·L^-1^ |  |
| Nicotinic acid | Duchefa |  |  |  | 2.5 mg·L^-1^ |  | 2 mg·L^-1^ | 2 mg·L^-1^ |  |
| Thiamine-HCl | Duchefa |  |  |  | 10 mg·L^-1^ |  | 1 mg·L^-1^ | 1 mg·L^-1^ |  |
| Pyridoxine-HCl | Duchefa |  |  |  | 1 mg·L^-1^ |  | 2 mg·L^-1^ | 2 mg·L^-1^ |  |
| Na-Pyruvate | Biochrom |  |  |  | 0.02 g·L^-1^ |  |  |  |  |
| Biotin | Amresco |  |  |  |  |  | 20 µg·L^-1^ | 20 µg·L^-1^ |  |
| Ca-pantothenate | Applichem |  |  |  |  |  | 2 mg·L^-1^ | 2 mg·L^-1^ |  |
| Citric acid | VWR |  |  |  | 0.04 g·L^-1^ |  |  |  |  |
| DL-Malic-acid | Duchefa |  |  |  | 0.04 g·L^-1^ |  |  |  |  |
| Fumaric acid | Sigma Aldrich |  |  |  | 0.04 g·L^-1^ |  |  |  |  |
| NH_4_Cl | Merck |  |  |  |  |  | 1.06 g·L^-1^ | 1.06 g·L^-1^ |  |
| KOH | Merck |  |  |  |  |  | 2.24 g·L^-1^ | 2.24 g·L^-1^ |  |
| Succinic acid | Roth |  |  |  |  |  | 2.36 g·L^-1^ | 2.36 g·L^-1^ |  |
| MES | Applichem |  |  |  |  | 0.195 g·L^-1^ | 0.195 g·L^-1^ | 0.195 g·L^-1^ |  |
| Mannitol | Duchefa |  |  |  | 0.25 g·L^-1^ | 85 g·L^-1^ |  |  |  |
| Fructose | Acros |  |  |  | 0.25 g·L^-1^ |  |  |  |  |
| Ribose | Duchefa |  |  |  | 0.25 g·L^-1^ |  |  |  |  |
| Xylose | Alfa Aesar |  |  |  | 0.25 g·L^-1^ |  |  |  |  |
| Mannose | Duchefa |  |  |  | 0.25 g·L^-1^ |  |  |  |  |
| Rhamnose | Millipore |  |  |  | 0.25 g·L^-1^ |  |  |  |  |
| Cellobiose | Fluka |  |  |  | 0.25 g·L^-1^ |  |  |  |  |

Media 1–4 are adjusted to pH 5.6, and media 5–7 to pH 5.8. The osmolarity of all media, except media 1 and 5, is adjusted to 560 mOsm kg⁻¹ H₂O using sucrose to ensure consistent osmotic conditions across experiments. Sucrose concentrations are therefore given as approximate values, as they were adjusted to reach the target osmolarity for each medium.

Supplementary Table S2. Estimated marginal means (EMMs) of relative protoplast number change across cultivation days (1, 3, 5, 7) and growth regulator concentrations (0, 20, 50, 80, and 150 µg·L⁻¹ BAP + NAA), with standard errors and 95% confidence intervals.


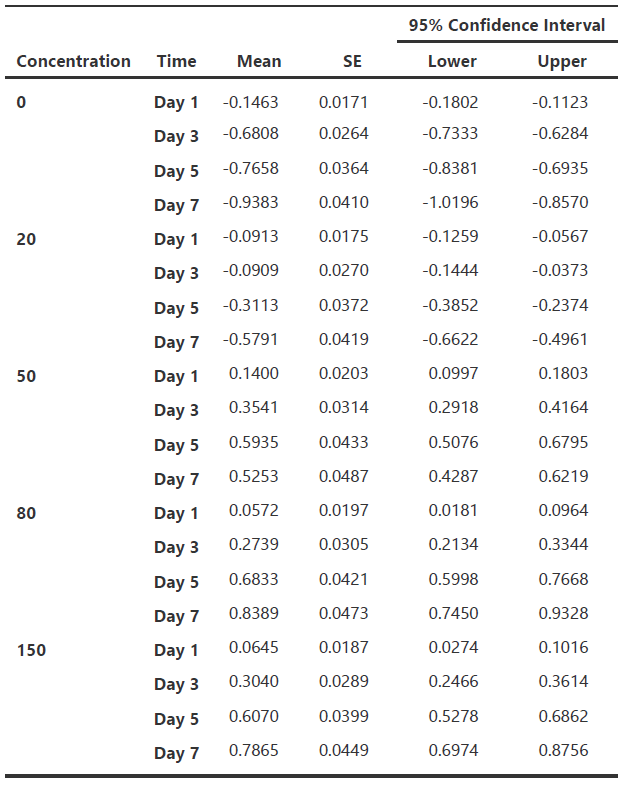


Supplementary Table S3. Estimated marginal means (EMMs) of relative protoplast diameter change across cultivation days (1, 3, 5, 7) and growth regulator concentrations (0, 20, 50, 80, and 150 µg·L⁻¹ BAP + NAA), with standard errors and 95% confidence intervals.


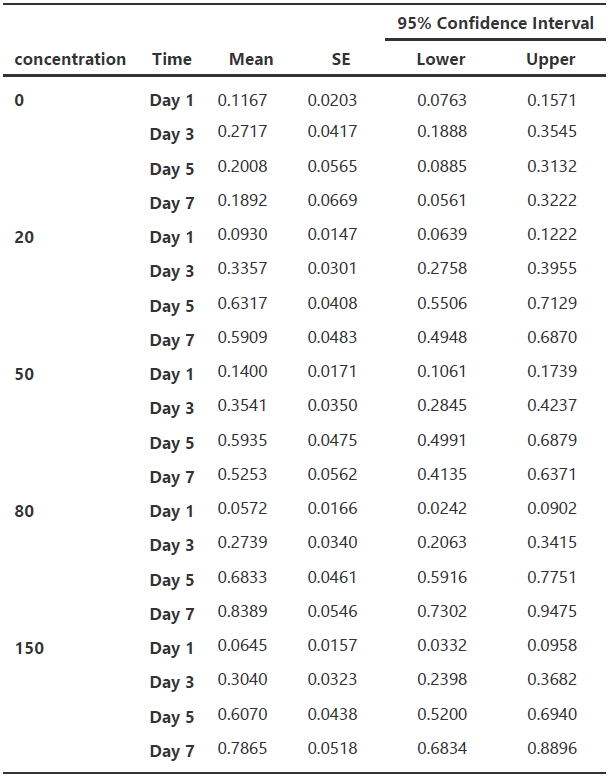

Supplement: Supplementary file 1 — Supplementary Material 1 [file 41598_2025_28956_MOESM1_ESM.docx]
